# ‘Diagnostic shock’: the impact of results from ultrarapid genomic sequencing of critically unwell children on aspects of family functioning

**DOI:** 10.1038/s41431-022-01140-8

**Published:** 2022-07-13

**Authors:** Hilary Bowman-Smart, Danya F. Vears, Gemma R. Brett, Melissa Martyn, Zornitza Stark, Christopher Gyngell

**Affiliations:** 1 Murdoch Children’s Research Institute, Melbourne, VIC Australia; 2 Department of Paediatrics, University of Melbourne, Melbourne, VIC Australia; 3 Ethox Centre, University of Oxford, Oxford, United Kingdom; 4 Victorian Clinical Genetics Services, Melbourne, VIC Australia; 5 Melbourne Genomics, Melbourne, VIC Australia; 6 Australian Genomics, Melbourne, VIC Australia

**Keywords:** Genetics research, Medical ethics

## Abstract

Rapid genomic sequencing (rGS) is being increasingly used in neonatal and paediatric intensive care units. While there is emerging evidence of clinical utility and cost-effectiveness, concerns have been raised regarding the impact of delivering genomic results in an acute care setting. To help investigate these concerns, we analysed survey data collected from caregivers whose children had received rGS through a national rapid genomic diagnosis program. The impact of rGS on families was assessed through the PedsQL2.0 Family Impact Module and the State-Trait Anxiety Inventory (STAI-6). Sixty-one parents/carers completed the survey during the study period (response rate 48%; 61/128). Mean parent and family functioning was reduced in this sample, reflecting the stressful conditions facing families with critically unwell children. We found caregivers whose children had received a diagnostic result through rGS reported a reduced family relationships score compared to caregivers of children who did not receive a diagnosis. These findings have implications for genetic counselling practice in this setting.

## Introduction

Rapid exome sequencing (rES) and rapid genome sequencing (rGS) (collectively known as genomic sequencing) are increasingly being integrated into neonatal and paediatric intensive care. Genomic sequencing is more effective at diagnosing rare diseases than other approaches, such as microarray, and can result in improved clinical management [1–3]. Previously, the time taken to generate results from genomic sequencing limited its role in acute care settings, and clinicians had to rely on other, sometimes invasive diagnostic tests. Rapid genomic sequencing offers the opportunity to bring the benefits that genomics has realised in the ambulatory setting into acute medical care. However, this shift brings with it unique challenges for genetic counselling, where processes may not have been developed to accommodate short timeframes to diagnosis [4].

Time is an essential factor when treating critically unwell children in neonatal and paediatric intensive care units (NICU and PICUs). A fast diagnosis may facilitate tailored, life-saving treatments [5, 6]. In cases where the diagnosis is associated with a poor prognosis, this knowledge may help reduce the number of painful investigations, and allow for re-direction to palliative care [5]. Even when rES/rGS does not lead to a diagnosis it can still positively impact clinical management by reducing the need for other investigations [7]. As is true for genomic sequencing generally, results from rES/rGS can be useful both for improving understanding of rare disease [8], and for allowing parents to make informed future reproductive decisions.

There is also emerging evidence of the cost-effectiveness of rES/rGS [9, 10], and its use in paediatric acute care is being trialled and/or implemented in multiple healthcare systems worldwide, including in the US [11–14], the UK [15], Australia [7], Canada [16], Hong Kong [17], China [18], the Netherlands [19], and Poland [20].

While there is evidence of clinical benefit, clinicians and bioethicists have raised a number of concerns around the use of rES/rGS in paediatric acute care [5]. One concern is that delivering a genomic diagnosis in the intensive care setting may be particularly disruptive for families. The newborn period is a crucial time for child-caregiver bonding and learning an infant has a life-long genetic condition during this period may be harder to process for caregivers, compared to when children are older [5, 21]. Results from testing may also lead to other negative psychosocial outcomes for both the parent(s) and the child, such as self or partner blame or detrimental impacts on family relationships [21].

## Context

To investigate whether such hypothetical concerns are realised in practice, data were drawn from a survey of parents of critically unwell children who were part of the Australian Genomics Health Alliance Acute Care Genomics study. The Acute Care Genomics study evaluated the feasibility of delivering ultra-rapid exome sequencing (urES) at scale in the Australian public healthcare system [7]. Paediatric patients with suspected monogenic conditions admitted to intensive care units across 12 hospitals underwent urES as a trio where possible; the median age was 28 days old (the majority being infants in the NICU) and the average turnaround time from sample receipt to sequencing report was three days [7]. A molecular diagnosis was established in 51% of the participants. urES results influenced clinical management in 76% of patients who received a diagnosis and 11% of those who did not receive a diagnosis (e.g. through ruling out certain genetic diagnoses) [7].

Survey data relating to parental experiences and outcomes were published, showing low decisional regret across all respondents, and higher empowerment in parents whose child received a genetic diagnosis [22]. Interviews with a subset of parents showed they found the process intensely stressful, yet they appreciated receiving access to a new technology [23]. To address potential ethical concerns relating to parent-child bonding, family relationships, and family functioning, here we report additional survey data relating to parental health and anxiety, as well as parent and family functioning.

## Subjects and methods

Survey invitations were sent to 128 families (one survey per family) via email >12 weeks after they had received the results of their child’s urES test; 97 received the full survey (those whose child was alive at time of invitation) and 31 received the shortened version (those whose child was deceased at time of invitation). Surveys collected from parents whose children underwent urES between March 2018 and February 2020 were included in the analysis. Survey invitations were not sent if parents showed signs of being in high distress as assessed by the clinical team (12 families), parents lacked sufficient English comprehension (15 families), or parents declined to leave contact details (12 families). Data were collected through the REDCap survey collection tool, hosted at the Murdoch Children’s Research Institute [24].

The survey used a combination of validated instruments and custom questions. The scales included in this analysis are a selection of scales from the PedsQL2.0 Family Impact Module, and the State-Trait Anxiety Inventory (STAI-6). These scales assessed the impact of the testing process on parent and family functioning, family relationships, and parental anxiety. Questions about parental health and relationships, and their perceptions of their child, were also included.

The PedsQL2.0 Family Impact Module was only shown to parents whose child was still alive at the time of survey invitation. Scores for this scale are out of 100, with a higher score indicating higher levels of parent and family functioning [25]. Parent functioning was assessed using two sub-scales (from a possible total of six in the module). The communication sub-scale (3 items) assesses how well parents feel they can communicate with other people (e.g., doctors). The worry sub-scale (5 items) assesses the level of worry parents have (e.g., about their child’s treatments, reactions of others, and their child’s future). Family functioning was assessed using two sub-scales: daily activities (3 items, covering problems with daily activities, such as household tasks) and family relationships (5 items, covering level of stress, conflict and communication difficulties between family members).

The STAI-6 was also only shown to parents whose child was still alive at the time of survey invitation. The STAI-6 assesses the level of state anxiety (how one feels at the time of completing the survey) [26]. Scores range from 20 to 80; scores ≥50 indicate elevated state anxiety.

Means are presented with standard deviations and differences in means are presented with 95% confidence intervals (CIs). Associations between the quantitative data were assessed using t-tests, linear regression, one-way ANOVA and χ^2^ or Fisher’s Exact tests (due to small sample size). Power calculations were performed to assess likely significance of observed results. Some variables – such as age of child at consent for urES or gross family income – were collapsed into binary variables for analysis; this is noted where applicable. Qualitative data from free-text responses were analysed by HBS and DV using content analysis [27].

## Results

Sixty-one caregivers completed the survey during the study period (response rate 48%; 61/128). Among the respondents, 66% (*n* = 40) of infants were alive at the time of survey invitation, with 34% (*n* = 21) deceased. Slightly more than half (*n* = 31, 51%) received a diagnosis. See Supplementary Table 1 for demographic information of survey respondents.

Some questions were not answered by all respondents, hence the total sample for each result is presented below.

### Altered thinking about the child (*n* = 60)

Most parents (*n* = 41, 68%) indicated that the rapid result had not altered their thinking about their child; 23% (*n* = 14) indicated that it had and 8% (*n* = 5) were unsure. Receiving a diagnosis was more likely to be associated with altered thinking about the child compared to no diagnosis (*p* = 0.001).

Comments provided in the free-text components of the survey give us some insights into the ways in which the rapid results altered parents’ thinking about their child. For one parent, the result forced them to acknowledge that the condition was genetic, and this fact changed their thinking. For other parents, the change in their thinking was positive; the result made them more confident in their child’s health or allowed them to think about what their child requires now and into the future. One parent indicated that *“…by ruling out a number of possibilities it has given me more opportunity to get to know & appreciate my baby without getting caught up in labels & diagnosis.”* [Respondent #11, child did not receive a diagnosis]. Two parents who reported altered thinking discussed both positive and negative impacts of the results.

“I did have a grief reaction and still have a big fear of the unknown as the prognosis can be quite poor… It did make me worry for his future, but also prepared me for challenges he may face, and how I can optimise his developmental potential.” [Respondent #30, child received a diagnosis]

Of the parents who stated the results had not altered their thinking of their child, many expressed that the nature of the results would not affect their love and/or support for their child.

“Waiting for results for <our son> did make us terrified for his future however we decided, no matter what, our son would write his own story and we would just be there to love and support him every step of the way.” [Respondent #15, child did not receive a diagnosis]

One of the parents from this group expressed mixed emotions about the impact of the information they received.

“I still wish we had the opportunity to prevent him entering this world and living the life we would have never hoped for him. However, knowing what his limitations are will ensure that we can provide better care for him now that he is here and there is no other choice.” [Respondent #1, child received a diagnosis]

### Parent and family functioning (PedsQL2.0 Family Impact Module) (*n* = 39)

**Fig. 1:**
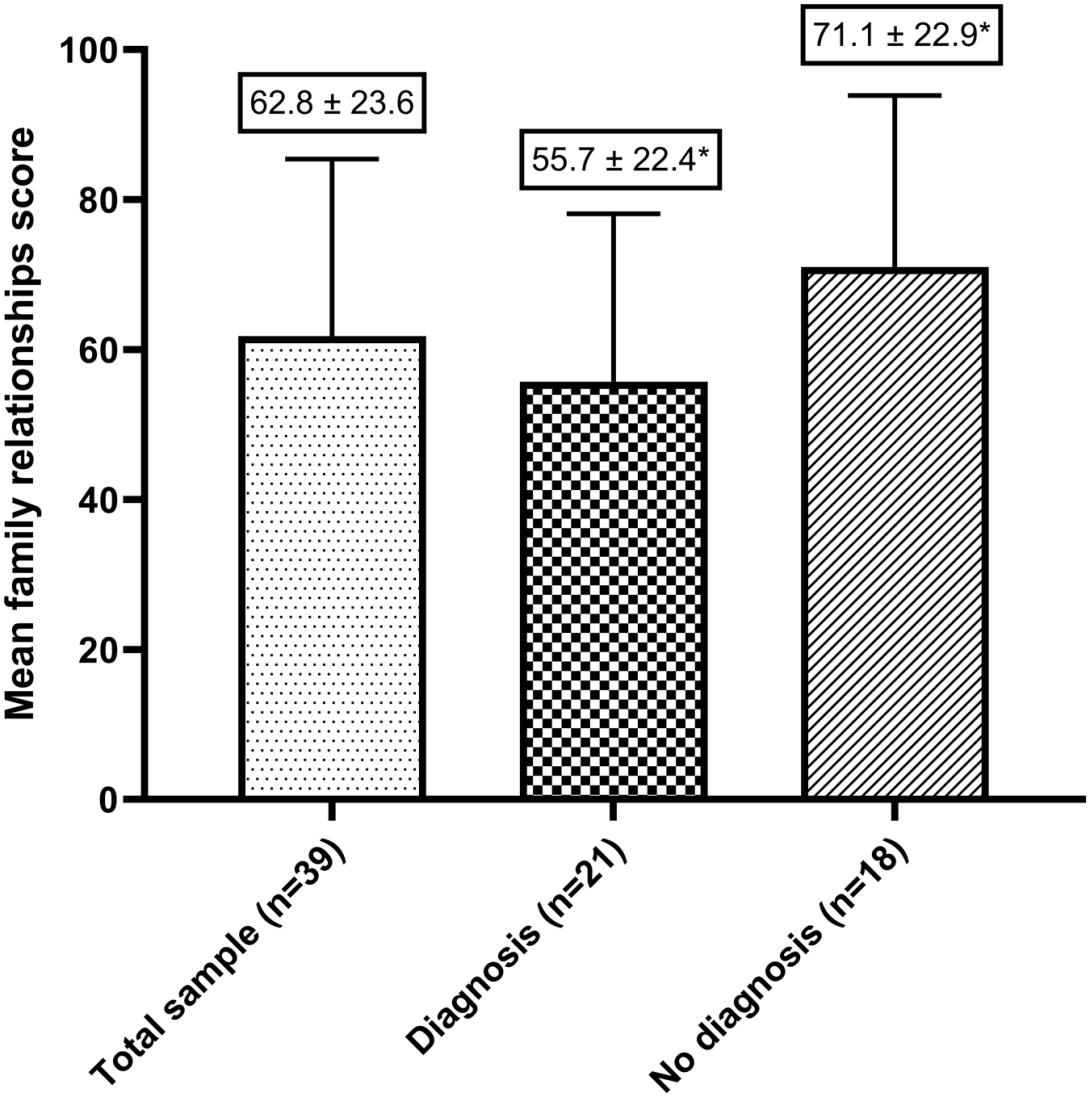
The mean family relationship sub-scores, categorised by whether urES resulted in a diagnosis. *Mean difference 15.5 points (95% CI: 0.8, 30.2), *p* = 0.039.

**Fig. 2:**
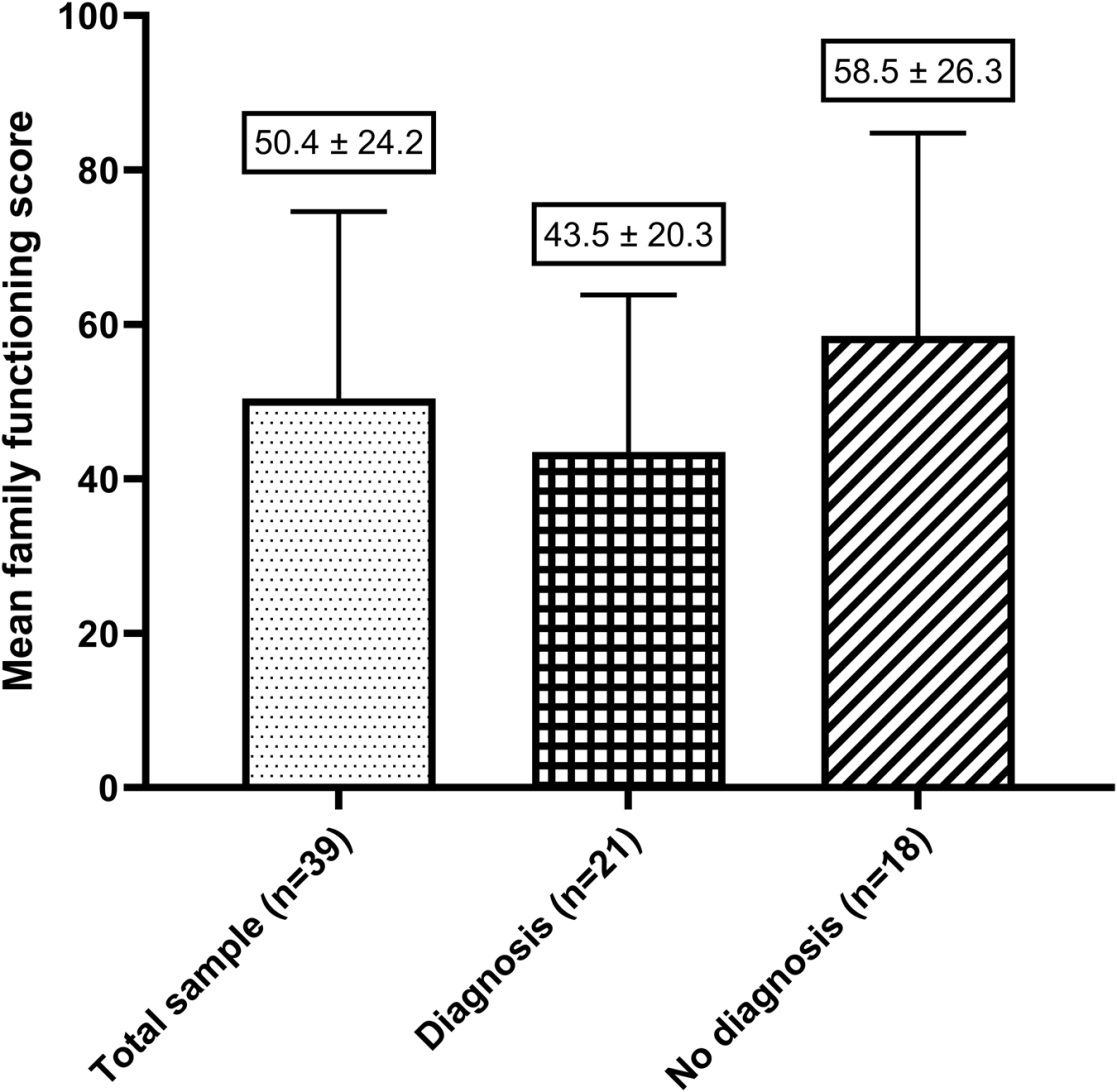
The mean family functioning scores, categorised by whether urES resulted in a diagnosis. Mean difference between groups is 15 points (95% CI: −0.2, 30.2), *p* = 0.0527.

**Fig. 3:**
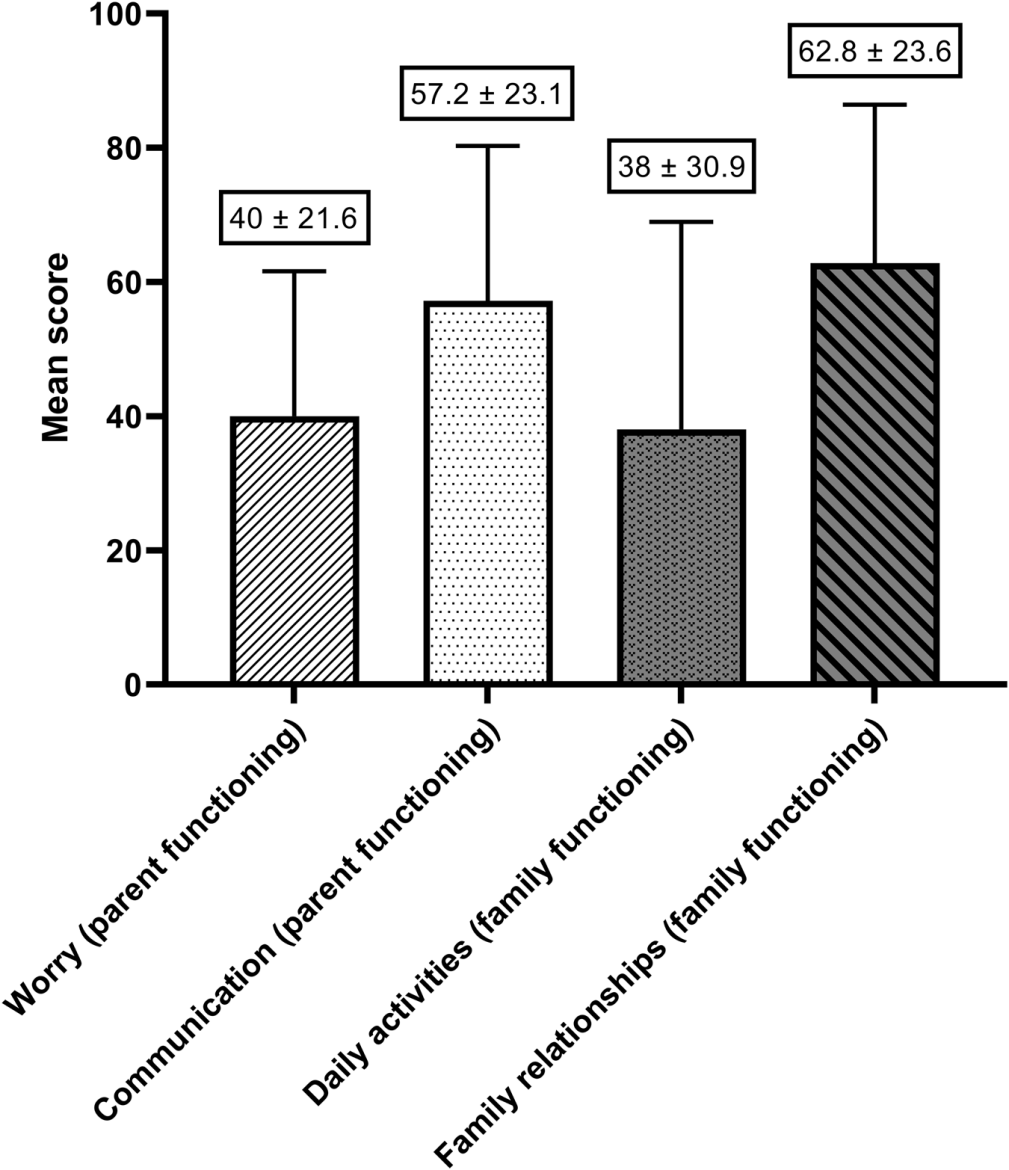
The mean scores across the four different scales of the PedsQL2.0 Family Impact Module that were assessed in this study (*n* = 39).

Those whose child received a diagnosis had a decreased family relationships score (a sub-score of family functioning) compared to those whose child did not receive a diagnosis, *p* = 0.039, Fig. 1. There were no associations between age of child at consent and Family Impact Module scores. The mean family functioning scores can be seen in Fig. 2, and the mean sub-scores in Fig. 3.

### State anxiety (STAI-6) (*n* = 39)

The mean score of respondents for the STAI-6 was 52 ± 13.1 out of 80. Most respondents (*n* = 26, 67%) had elevated state anxiety.

Increased levels of state anxiety were significantly correlated with increased levels of worry (*r*^2^ = 0.39, *p* < 0.0001) and problems with communication (*r*^2^ = 0.35, *p* = 0.0001) as assessed by the PedsQL2.0 Family Impact Module sub-scores. Increased levels of state anxiety were also slightly correlated with decreased family functioning (*r*^2^ = 0.21, *p* = 0.0031). There was no association between state anxiety and the child’s result.

### Parent relationships and health

**Fig. 4:**
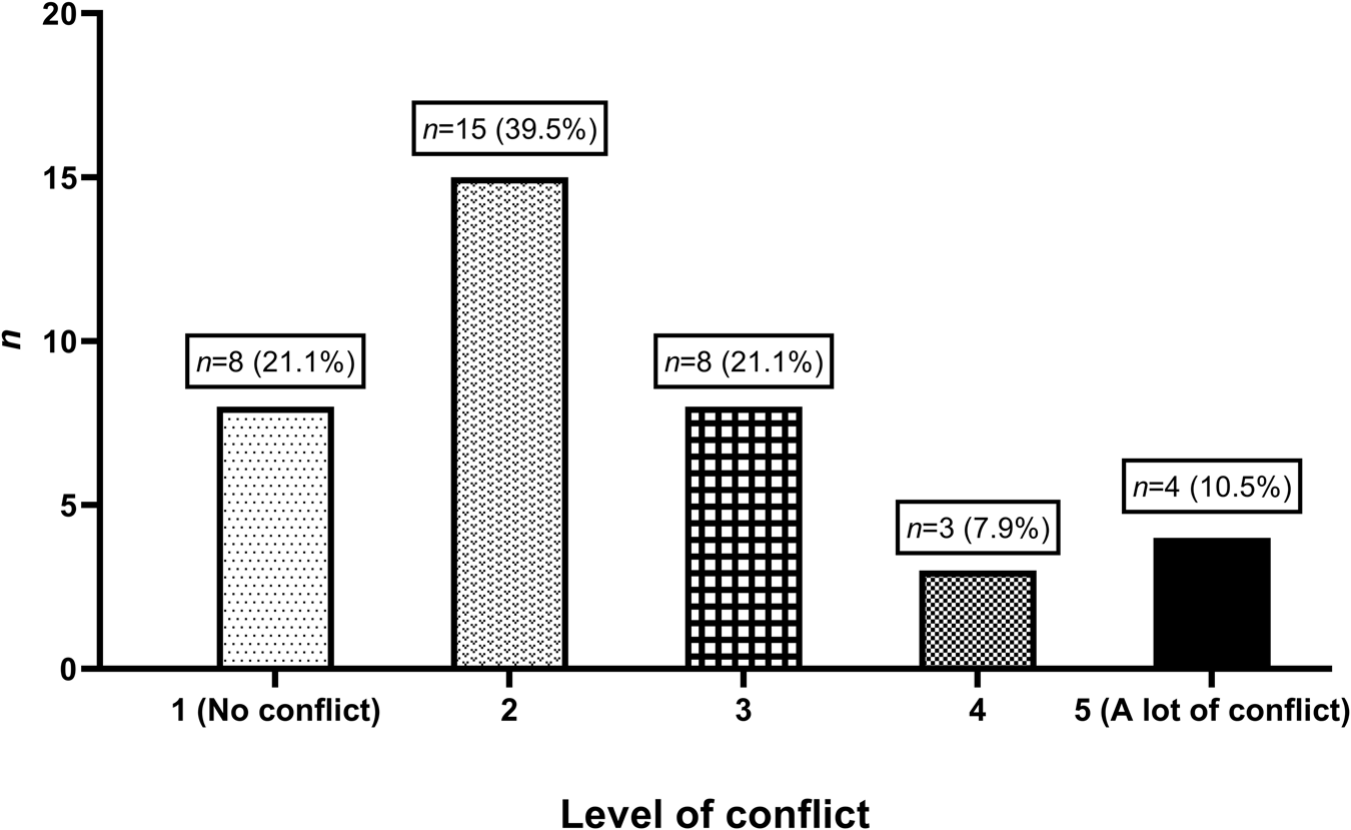
The degree of conflict with their partners our cohort reported experiencing (*n* = 38).

**Fig. 5:**
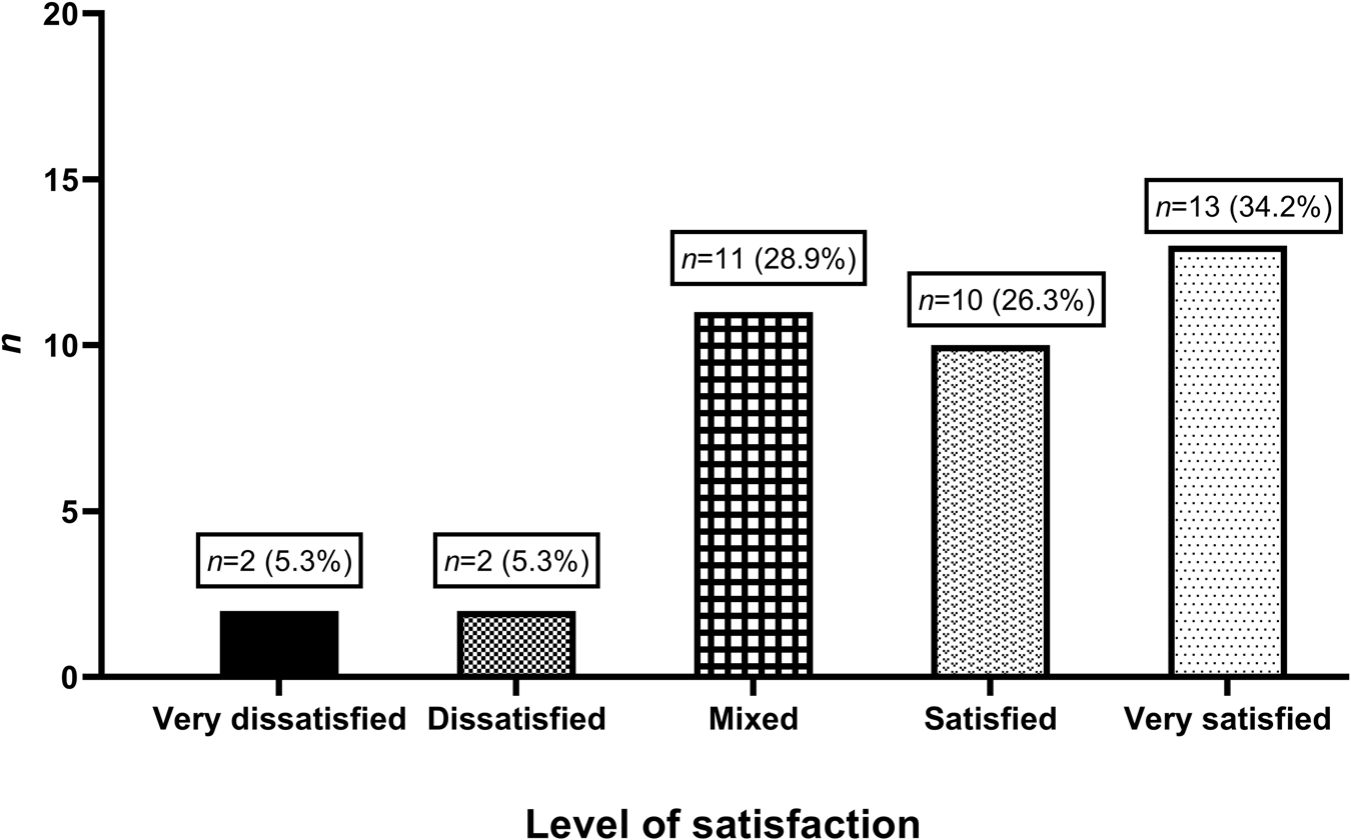
The level of satisfaction with their relationship with their partners our cohort reported experiencing (*n* = 38).

Parents generally indicated they experienced low levels of conflict with their partner (Fig. 4), with over 60% (23/38) indicating they experienced no or little conflict, and just over 10% (4/38) indicating they experienced ‘a lot of conflict’. Similarly, most parents indicated satisfaction with their relationship with their partner (Fig. 5), with 61% (23/38) being either ‘very satisfied’ or ‘satisfied’. There was no association between spousal conflict and the child’s result.

Parents (*n* = 34) were asked to rank ‘how good or bad your health has been over the past 4 weeks’ on a scale of 0–100, with 100 being the ‘best health state you can imagine’ and 0 being the worst. Participants were not guided whether this rating should be specific to physical health, mental health and/or general wellbeing. The mean score for these respondents was 68 ± 21 out of 100. Spousal satisfaction was positively associated with parent health (*p* = 0.0124). The mean parent health score for those who were ‘very satisfied’ with their relationship with their partner was 74.3 ± 21, compared to 25 ± 7.1 for those who were ‘very dissatisfied’.

## Discussion

The aim of this study was to explore concerns about the possible effects of urES in the paediatric acute care setting on family relationships and functioning. We found that receiving a diagnosis through urES is associated with a decrease in aspects of family functioning and parents having altered thinking about their child. Observations from this study are discussed below in the context of potential clinical implications and limitations.

Our analysis found a significant association between receiving a diagnosis from urES and a decreased family relationships score. Research in other settings has found that diagnosing children with an illness is associated with significant reductions in family functioning and problems with family relationships [28]. Previous studies have used a comparison between family functioning, including family relationships, in children diagnosed with an illness versus healthy controls [29]. However, these studies cannot separate the effects on family relationships and functioning of caring for a sick child versus the effects of actively receiving a specific diagnosis. This leaves open the question of how receiving a diagnosis affects family relationships and functioning, independently from the effects of caring for a sick child. Our analysis helped explore this question in an acute care setting. As only sick children were considered for urES, and roughly half received a diagnosis, the data collected for our study potentially provides a way to distinguish the effects of receiving a diagnosis on family  relationships from the effects of caring for a critically unwell infant. As our analysis found an association between receiving a diagnosis and a decreased family relationships score, it provides tentative evidence that, in an acute care setting, additional strains on aspects of family functioning may be caused by receiving a diagnosis, above that of caring for a sick child. One possible reason for the difference in attitudes between parents who received a diagnosis versus those who did not, is that receiving a diagnosis may raise further questions and uncertainties and necessitate further actions and decisions. This may make it harder for parents to accept and give meaning to their situations (we thank an anonymous reviewer for this suggestion). Further research is required to confirm this association, including whether it is observed in other settings, and for non-genomic diagnoses.

Our analysis provides some evidence regarding an emerging concern for rES/rGS; that genomic results may lead to negative impacts on child-caregiver bonding. Two related, but separate, concerns have been expressed in the literature. The first is that receiving a genomic diagnosis early in the newborn period may be particularly distressing at the time when results are returned because there has not been sufficient time for caregivers and children to bond [5]. We investigated this concern within this cohort by comparing family functioning (including family relationships) and the age of the child when caregivers provided consent for testing. If bonding mediates the impact of receiving results through rES/rGS, then receiving a diagnosis when a child is days or weeks old (when there has been little opportunity for bonding), should be more disruptive than receiving a diagnosis when a child is months or years old. Our study found no associations between any scores from the Family Impact Module and age of child at consent; therefore, we found no support for the idea that returning rES/rGS results in the newborn period is more disruptive than when children are older. However, it remains possible that an association between age of child at testing and impact on family functioning would be detected in a larger sample.

The second concern relating to rES/rGS and bonding is that receiving a genomic diagnosis through rES/rGS may in general disrupt bonding and have negative impacts on children and families going forward [30]. The fact that we found an association between a decreased family relationships score and receiving a diagnosis through urES may support this concern, as reduced quality of family relationships may disrupt bonding. However, it is not clear that the association we found between receiving a diagnosis and a decreased family relationships score has a persistent effect on relationships. Pereira et al. [31], found no persistent negative psychosocial effect on children and families who underwent rGS, compared to other children and families in the NICU who did not undergo rGS.

One reason why aspects of family functioning may be reduced in parents who receive a diagnosis is that it leads to heightened anxiety in caregivers and increased spousal conflict. However, our analysis found no association between receiving a diagnosis through urES and spousal conflict or state anxiety. An alternative explanation is that receiving a genomic diagnosis changes caregivers’ perceptions of their child, and this in turn could reduce aspects of family functioning. We did find that receiving a diagnosis through urES was associated with caregivers having altered thinking about their child. This altered thinking may take a variety of forms depending on the nature of the result provided by urES. In some cases a diagnosis may remove parents’ hope that their child will be completely ‘cured’, and that these current challenges are temporary. Some of the qualitative responses received support this interpretation, with respondents reporting feeling fear for their child’s future. Furthermore, in non-acute settings, the process of receiving news about a diagnosis of a genetic condition in a child often involves parents transitioning from ‘watchful waiting’ to ‘acceptance’ [32] over a period of months or years. However, for many of the caregivers who participated in our study, their ‘watchful waiting’ period would have been minimal, and the lack of this adjustment period may have contributed to any negative impact of receiving a diagnosis/result. While a sudden diagnosis or illness can occur in many areas of medicine, the very young age and rapid time to result in this context might contribute to some parents experiencing what we call ‘diagnostic shock’.

However, altered thinking can also take positive forms. Caregivers in our cohort described how finding a cause for their child’s condition may have allowed them to accept the illness, envision a path forward, and understand their child’s needs. Furthermore, other research has indicated that parents generally respond positively to receiving results from rapid genomic sequencing. Hill et al. report that in a sample where the median age of the child was 2.5 months, parents viewed a diagnosis from rapid genomic sequencing positively [30]. Cakici et al. found similar results in their study where the median age of infants was four days, and also that parents who received a diagnosis were more likely to perceive the results as useful [33]. The qualitative responses recorded in our study illustrate the diverse ways in which parents make meaning in response to rapid genomic results.

Although caregivers in this cohort reported low decisional regret [22], our analysis creates a more detailed picture of what a family might experience and what additional supports might be required. The observation that aspects of family functioning is low in groups receiving a diagnosis through urES in paediatric acute care may help inform genetic counselling practice in this and other settings, with regard to the potential need for additional counselling and/or supports to be offered to parents who receive a diagnosis in their child via genomic testing [34]. Furthermore, caregivers may benefit from increased support and counselling; genetic counsellors could alert parents during pre-test counselling to the potential impacts of this experience on their family relationships, and also explore parent feelings and relationships post-test to identify and recommend appropriate external and longer-term supports. While it may not be possible nor practical to discuss all potential outcomes of genomic testing during pre-test counselling in acute care settings, this study emphasises the value in exploring and managing parental expectations.

## Limitations

Although being critically unwell was a requirement for all children in this study to receive urES, it is not clear whether the group of children who received a diagnosis were equally as unwell as children who did not receive a diagnosis at the time the survey was completed. Some children in the group who did not receive a diagnosis had transient problems which had resolved by the time caregivers completed the survey. Conversely, some of the children for whom a diagnosis was received were able to be offered better treatment plans based on their diagnosis. It could be that underlying differences in average health, or differences in clinical management, between the two groups contributed to differences in family relationships scores.

Furthermore, we did not separate families where a diagnosis defined a treatment pathway for the child, versus those where a diagnosis did not define a treatment pathway. The impact of a diagnosis on family relationships and parent and family functioning may be different in those groups, and larger cohorts will be necessary to determine this.

Further limitations of this study include the small sample size and restricted eligibility criteria, in particular the restricted application of some elements of the survey. Parents who were experiencing high distress (as assessed by the clinical team), who did not have sufficient English comprehension, or who did not leave contact details, were not sent a survey invitation. This may also have contributed to selection bias and impacted our findings. Further research on these phenomena in the excluded demographic groups, with a larger sample size would be useful for expanding upon our findings.

## Conclusions

This exploratory study provides important insight into the possible effects of urES in the paediatric acute care setting on family relationships and functioning. The findings have implications for genetic counselling practice and future research. The results from this study suggest that healthcare professionals involved in the provision of urES in paediatric acute care should be aware of the possibility of ‘diagnostic shock’, and provide families appropriate pre-test counselling with extra psychological support following result return. These data also support previous work outlining the challenges faced in genetic counselling practice in acute care settings compared to traditional ambulatory settings [4, 34, 35]. Importantly, health professionals such as genetic counsellors need to be aware of the potential for family relationships to be impacted and/or parents to have altered (positive or negative) thinking about their child after receiving a genetic diagnosis in their child.

## Supplementary information

Sup Table 1

## Data Availability

Data generated as part of this study are available from the corresponding author on reasonable request.

## References

[1] Stavropoulos DJ, Merico D, Jobling R, Bowdin S, Monfared N, Thiruvahindrapuram B (2016). Whole-genome sequencing expands diagnostic utility and improves clinical management in paediatric medicine. NPJ Genom Med.

[2] Lionel AC, Costain G, Monfared N, Walker S, Reuter MS, Hosseini SM (2018). Improved diagnostic yield compared with targeted gene sequencing panels suggests a role for whole-genome sequencing as a first-tier genetic test. Genet Med.

[3] Clark MM, Stark Z, Farnaes L, Tan TY, White SM, Dimmock D (2018). Meta-analysis of the diagnostic and clinical utility of genome and exome sequencing and chromosomal microarray in children with suspected genetic diseases. npj Genom Med.

[4] Lynch F, Nisselle A, Gaff CL, McClaren B (2021). Rapid acute care genomics: challenges and opportunities for genetic counselors. J Gen Couns..

[5] Gyngell C, Newson AJ, Wilkinson D, Stark Z, Savulescu J (2019). Rapid challenges: ethics and genomic neonatal intensive care. Pediatrics..

[6] Bowdin S, Gilbert A, Bedoukian E, Carew C, Adam MP, Belmont J (2016). Recommendations for the integration of genomics into clinical practice. Genet Med.

[7] Australian Genomics Health Alliance Acute Care Flagship. (2020). Feasibility of ultra-rapid exome sequencing in critically ill infants and children with suspected monogenic conditions in the Australian public health care system. JAMA..

[8] French CE, Delon I, Dolling H, Sanchis-Juan A, Shamardina O, Mégy K (2019). Whole genome sequencing reveals that genetic conditions are frequent in intensively ill children. Intens Care Med.

[9] Farnaes L, Hildreth A, Sweeney NM, Clark MM, Chowdhury S, Nahas S (2018). Rapid whole-genome sequencing decreases infant morbidity and cost of hospitalization. Genom Med..

[10] Stark Z, Lunke S, Brett GR, Tan NB, Stapleton R, Kumble S (2018). Meeting the challenges of implementing rapid genomic testing in acute pediatric care. Genet Med.

[11] Petrikin JE, Cakici JA, Clark MM, Willig LK, Sweeney NM, Farrow EG (2018). The NSIGHT1-randomized controlled trial: rapid whole-genome sequencing for accelerated etiologic diagnosis in critically ill infants. NPJ Genom Med.

[12] Dimmock D, Caylor S, Waldman B, Benson W, Ashburner C, Carmichael JL (2021). Project Baby Bear: Rapid precision care incorporating rWGS in 5 California children’s hospitals demonstrates improved clinical outcomes and reduced costs of care. Am J Hum Genet.

[13] Kingsmore SF, Cakici JA, Clark MM, Gaughran M, Feddock M, Batalov S (2019). A randomized, controlled trial of the analytic and diagnostic performance of singleton and trio, rapid genome and exome sequencing in ill infants. Am J Hum Genet.

[14] Gubbels CS, VanNoy GE, Madden JA, Copenheaver D, Yang S, Wojcik MH (2020). Prospective, phenotype-driven selection of critically ill neonates for rapid exome sequencing is associated with high diagnostic yield. Genet Med.

[15] Mestek-Boukhibar L, Clement E, Jones WD, Drury S, Ocaka L, Gagunashvili A (2018). Rapid Paediatric Sequencing (RaPS): Comprehensive real-life workflow for rapid diagnosis of critically ill children. J Med Genet.

[16] Elliott AM. Genetic counseling and genome sequencing in pediatric rare disease. Cold Spring Harbor Pers Med. 2019;10:a036632. 10.1101/cshperspect.a036632 PMC7050583 31501267

[17] Chung CC, Leung GK, Mak CC, Fung JL, Lee M, Pei SL (2020). Rapid whole-exome sequencing facilitates precision medicine in paediatric rare disease patients and reduces healthcare costs. Lancet Regional Health West Pac.

[18] Wang H, Lu Y, Dong X, Lu G, Cheng G, Qian Y (2020). Optimized trio genome sequencing (OTGS) as a first-tier genetic test in critically ill infants: practice in China. Hum Genet.

[19] van Diemen CC, Kerstjens-Frederikse WS, Bergman KA, de Koning TJ, Sikkema-Raddatz B, van der Velde JK (2017). Rapid targeted genomics in critically ill newborns. Pediatrics..

[20] Śmigiel R, Biela M, Szmyd K, Błoch M, Szmida E, Skiba P (2020). Rapid whole-exome sequencing as a diagnostic tool in a neonatal/pediatric intensive care unit. J Clin Med.

[21] Frankel LA, Pereira S, McGuire AL. Potential psychosocial risks of sequencing newborns. Pediatrics. 2016;137:S24–9. 10.1542/peds.2015-3731F PMC9923971 26729699

[22] Brett GR, Martyn M, Lynch F, de Silva MG, Ayres S, Gallacher L (2020). Parental experiences of ultrarapid genomic testing for their critically unwell infants and children. Genet Med..

[23] Lynch F, Nisselle A, Stark Z, Gaff CL, McClaren B. Parents’ experiences of decision making for rapid genomic sequencing in intensive care. EJHG. 2021;29:1804–10. 10.1038/s41431-021-00950-6 PMC8632931 34426661

[24] Harris PA, Taylor R, Thielke R, Payne J, Gonzalez N, Conde JG (2009). Research electronic data capture (REDCap)—a metadata-driven methodology and workflow process for providing translational research informatics support. J Biomed Inf.

[25] Varni JW, Sherman SA, Burwinkle TM, Dickinson PE, Dixon P (2004). The PedsQL™ family impact module: preliminary reliability and validity. Health Qual Life Out.

[26] Marteau TM, Bekker H (1992). The development of a six-item short-form of the state scale of the Spielberger State—Trait Anxiety Inventory (STAI). Brit J Clin Psych.

[27] Downe‐Wamboldt B (1992). Content analysis: method, applications, and issues. Health Care Women Int.

[28] Pai AL, Greenley RN, Lewandowski A, Drotar D, Youngstrom E, Peterson CC (2007). A meta-analytic review of the influence of pediatric cancer on parent and family functioning. J Fam Psych..

[29] McClellan CB, Cohen LL (2007). Family functioning in children with chronic illness compared with healthy controls: a critical review. J Ped..

[30] Hill M, Hammond J, Lewis C, Mellis R, Clement E, Chitty LS (2020). Delivering genome sequencing for rapid genetic diagnosis in critically ill children: parent and professional views, experiences and challenges. EJHG.

[31] Pereira S, Smith HS, Frankel LA, Christensen KD, Islam R, Robinson JO (2021). Psychosocial effect of newborn genomic sequencing on families in the BabySeq Project: A randomized clinical trial. JAMA Pediatrics..

[32] Ashtiani S, Makela N, Carrion P, Austin J (2014). Parents’ experiences of receiving their child’s genetic diagnosis: a qualitative study to inform clinical genetics practice. Am J Med Genet.

[33] Cakici JA, Dimmock DP, Caylor SA, Gaughran M, Clarke C, Triplett C (2020). A prospective study of parental perceptions of rapid whole-genome and -exome sequencing among seriously ill infants. Am J Hum Genet.

[34] Ayres S, Gallacher L, Stark Z, Brett GR (2019). Genetic counseling in pediatric acute care: reflections on ultra‐rapid genomic diagnoses in neonates. J Gen Couns..

[35] Diamonstein CJ (2019). Factors complicating the informed consent process for whole exome sequencing in neonatal and pediatic intensive care units. J Gen Couns..

