# Supplementary material for: ‘Diagnostic shock’: the impact of results from ultrarapid genomic sequencing of critically unwell children on aspects of family functioning
Source: Eur J Hum Genet. 2022 Jul 13;30(9):1036–43. doi: 10.1038/s41431-022-01140-8 (PMC9436940; doi:10.1038/s41431-022-01140-8)
Supplement: Supplementary file 1 — Sup Table 1 [file 41431_2022_1140_MOESM1_ESM.docx]

**Supplementary table 1: Demographic information**

| **Variable** | **Number of Respondents (%)** |
| --- | --- |
| **Age of patient** |  |
| 0 months | 27 (44) |
| 1 to 5 months | 20 (33) |
| 6 to 11 months | 6 (10) |
| 12 to 17 months | 0 (0) |
| 18 months up to 5 years | 4 (7) |
| 5 years up to 10 years | 3 (5) |
| 10 years and over | 1 (2) |
| **Relationship of survey respondent to patient** |  |
| Mother | 48 (80) |
| Father | 10 (17) |
| Other | 2 (3) |
| **Gender of survey respondent** |  |
| Male | 10 (17) |
| Female | 50 (83) |
| Other | 0 (0) |
| **Age of survey respondent (n=61)** |  |
| Under 25 | 2 (3) |
| 25-29 | 12 (20) |
| 30-34 | 21 (36) |
| 35-39 | 16 (27) |
| 40 and over | 8 (14) |
| **Relationship status of survey respondent** |  |
| De facto (living with a partner) | 12 (20) |
| Married | 43 (72) |
| Divorced/separated | 2 (3) |
| Never married | 2 (3) |
| Other | 1 (2) |
| **Education level of survey respondent** |  |
| Secondary | 9 (15) |
| Post-secondary | 51 (85) |
| **Number of children of survey respondent** |  |
| 0 | 11 (18) |
| 1 | 29 (48) |
| 2 | 14 (23) |
| 3 | 5 (8) |
| 4 | 0 (0) |
| 5 | 1 (2) |
| **Household income of survey respondent** |  |
| Less than $30,000 | 5 (9) |
| $30,000 - $59,999 | 6 (11) |
| $60,000 - $89,999 | 13 (22) |
| $90,000 - $119,999 | 11 (19) |
| $120,000 - $149,999 | 9 (16) |
| $150,000 or more | 9 (16) |
| Prefer not to answer | 4 (7) |
